# Addressing Residual Challenges of VISION 2020: The Right to Sight

**DOI:** 10.4103/0974-9233.80693

**Published:** 2011

**Authors:** Olufemi E. Babalola

**Affiliations:** Department of Ophthalmology, Rachel Eye Center, Abuja, Nigeria

The objective of VISION 2020: The Right to Sight is to create a world in which needless blindness is eliminated and those with unavoidable visual loss can achieve their full potential by the year 2020. This objective can only be achieved by integrating sustainable, comprehensive, and equitable eye care in national healthcare systems. The tenets of VISION 2020 are development of human resources, development of infrastructure, disease control, and proactive advocacy. In 1990,^1^ projections indicated that if the trends in avoidable vision loss and blindness continued unabated, there would be a doubling of avoidable vision impairment by 2020.

The main target diseases in the first phase of VISION 2020 are cataracts, trachoma, onchocerciasis, childhood blindness (particularly xerophthalmia and vitamin A deficiency), and uncorrected refractive errors. Glaucoma is now regarded as a sort of “missing link” and VISION 2020 India successfully advocated for the inclusion of glaucoma in the next phase by the Indian government.^2^–^4^

This edition of MEAJO is dedicated to Public Health issues of import to VISION 2020 challenges, particularly within Africa and the Middle East. Significant progress has been made since the inception of the program, and some notable success has been recorded in some key areas. Thylefors *et al*.^1^ estimated the prevalence of blindness worldwide at 38 million by 1990. Of note, the World Health Organization estimates the current prevalence of blindness globally at 39 million.^5^ This is particularly encouraging as it suggests that 16 years since Thylefors *et al*.’s estimates, this grim statistic is not increasing precipitously as originally forecasted-—in fact, it might be getting better. For example, trachoma accounts for approximately 4% of global blindness, which is an 8% decrease from some decades ago (as outlined by Rabiu *et al*., in this issue). In Ghana, trachoma is no longer a disease of public health importance, and other countries report similar progress.^6^ Despite these achievements, numerous challenges remain for controlling trachoma control in some endemic communities.

The control of onchocerciasis is no less impressive. The significant reduction of transmission of onchocerciasis in most of the Volta river basin has resulted in a cessation of the Onchocerciasis Control Program that was initiated in many regions of West Africa. Successor programs for the distribution of ivermectin have also been very successful in the African Program Onchocerciasis Control countries and the Onchocerciasis Elimination Program for the Americas countries in central and South America. New cases of onchocercal blindness are no longer reported in South America and are very rare in Africa.

Progress also continues in controlling cataract-related blindness. In this issue, Adel Rushood and colleagues report that the Al-basir foundation has been carrying out subsidized surgery in many countries in the Middle East, Asia, and some parts of Africa. More than two million people were examined and/or treated and almost 200 000 surgeries performed.

Despite these successes, however, challenges remain. In Sokoto State, Nigeria, the prevalence of bilateral blindness is approximately 2%, over half of which was due to cataracts. Unfortunately, however, the surgical coverage for cataract was lower than the couching coverage (4.4%*vs*14.9%, respectively).

A seemingly underestimated cause of blindness is ocular injuries. Three articles address ocular trauma in this issue, including one from the volatile Palestinian region.

Compounding the situation is the fact that research may not be well funded and/or may be of low quality in several developing countries as document by Mahmoud and colleagues in this issue.

In addition, public health strategies must evolve quickly to address “newly emergent” disease, particularly diabetic eye disease, age-related macular degeneration, and retinopathy of prematurity. Childhood blindness in general remains a continuing challenge, particularly training of health personnel (Gogate and colleagues). Courtright and colleagues address the issue of gender inequity in access to eye health, particularly in developing counties in sub-Saharan Africa.

There is therefore no room for complacency; we must be vigilant, an “all hands on deck” attitude needs to prevail to address the remaining challenges of VISION 2020. We have 9 years to go.
